# Factors Determining Retreatment Time Interval of Rituximab in Korean Patients With Rheumatoid Arthritis

**DOI:** 10.3389/fmed.2021.765535

**Published:** 2021-10-28

**Authors:** Ji-Won Kim, Ju-Yang Jung, Kichul Shin, Chang-Hee Suh, Hyoun-Ah Kim

**Affiliations:** ^1^Department of Rheumatology, Ajou University School of Medicine, Suwon, South Korea; ^2^Division of Rheumatology, Seoul Metropolitan Government-Seoul National University Boramae Medical Centre, Seoul, South Korea

**Keywords:** rituximab, rheumatoid arthritis, treatment response, adverse event, safety

## Abstract

Unlike other biologic agents for rheumatoid arthritis (RA) that are administered at regular intervals even without flare, rituximab can be administered according to the timing of retreatment determined by the physician. Recently, there has been a tendency to prefer on-demand administration for disease flares rather than regular retreatment. We aimed to investigate the retreatment patterns of rituximab in patients with RA and to identify factors associated with extension of the time interval between retreatment courses. This study included RA patients on rituximab treatment who were enrolled in the Korean Rheumatology Biologics registry (KOBIO) or treated at Ajou University Hospital. Previous or current concomitant conventional synthetic disease-modifying anti-rheumatic drugs (csDMARDs), corticosteroids, number of previous biologic agents, withdrawal, and time intervals of rituximab retreatment were collected. In case of treatment failure, the reasons such as lack of efficacy, adverse events, and others, were also identified. A total of 82 patients were enrolled. The mean follow-up period from the first cycle of rituximab was 46.1 months, and the mean interval between the retreatment courses was 16.3 months. The persistent rates of rituximab after 5 years was 72.4%. Concomitant use of at least two csDMARDs (β = 4.672; 95% CI: 0.089–9.255, *p* = 0.046) and concomitant use of corticosteroids (β = 7.602; 95% CI: 0.924–14.28, *p* = 0.026) were independent factors for extending the time interval between the retreatment courses. In conclusion, RA patients treated with rituximab in Korea show high persistence rates. Concomitant use of two or more csDMARDs and concomitant use of corticosteroids with rituximab are associating factors of extending the retreatment time interval. These findings should be considered when selecting rituximab as a treatment for patients with RA.

## Introduction

Rheumatoid arthritis (RA) is a chronic systemic inflammatory disorder that primarily affects the synovial joints. It is characterised by joint pain and functional disability that lead to reduced quality of life and a high socioeconomic burden (1). As an autoimmune disease, complex interactions among B cells, T cells, macrophages, neutrophils, dendritic cells, fibroblasts, and osteoclasts play crucial roles in initiating and maintaining inflammation of the joints (2). Among these, B cells appear to contribute significantly to the development of RA by producing pathogenic autoantibodies, presenting self-antigens to T cells, and secreting inflammatory cytokines (3). Therefore, B cells have emerged as therapeutic targets in treatment approaches involving direct depletion through monoclonal antibodies (mAb), inhibition of pro-inflammatory soluble factors or co-stimulatory molecules, and interruption of B cell activation or engagement of inhibitory checkpoint receptors (4). Despite the development of B cell-targeted treatment, the first therapeutic anti-CD20 mAb remains a crucial modality, with a long history of successful clinical use. In RA, the only biological agent approved for specific B cell-targeted therapy is rituximab, a chimeric monoclonal antibody against the CD20 antigen of B cells (5).

Rituximab was first used as a treatment for RA in 2006 and was approved for use with methotrexate (MTX) in patients with inappropriate responses to more than one anti-tumour necrosis factor agent (6). Clinical studies of rituximab have established its efficacy and safety as a protocol administered every 6 months after baseline. As such, the authorised dose regimen is intravenous infusion of 1,000 mg on days 1 and 15 every 24 weeks (7). However, although administration at intervals of 6 months may be the most appropriate, clinical responses may vary depending on seropositivity, biomarkers, and genetic markers given that reconstitution of the peripheral B cells usually occurs 6–9 months after rituximab administration (8, 9). Thus, determining the optimal timing for retreatment is challenging with respect to the duration of the effect. Further, safety is also of primary concern as repeated administration of rituximab may cause immunoglobulin reduction and increase the risk of infection. A comparison of the clinical effects of administration at reduced doses showed similar clinical effects at a reduced dose (one infusion of 1,000 mg or two doses of 500 mg) after a first course of rituximab at standard doses (10, 11). In addition, a recent real-world study on the use of rituximab showed that on-demand administration maintains good clinical responses (12).

Retreatment options for rituximab include regular retreatment at fixed intervals (e.g., every 6 months), treatment of flare, or treatment with any deterioration or treatment-to-target (13). This study aimed to investigate the patterns of use of rituximab in patients with RA in the real world, using the KOrean Rheumatology BIOlogics registry (KOBIO). Furthermore, we analysed the persistence rates of rituximab treatment and identified the factors associated with extending the administration intervals and with treatment failure.

## Methods

### Study Design and Population

The KOBIO registry is a nationwide, multi-centre, prospective, observational cohort formed by the Korean College of Rheumatology Biologics Registry and launched in 2012 (14). The KOBIO RA registry consisted of the biologic group and the control group [(patients treated with conventional synthetic disease-modifying anti-rheumatic drugs (csDMARDs)]. The biologic group involved patients aged over 18 years who have been diagnosed with RA and are initiating, restarting, or changing to a new biologic agent. The purpose of the registry was to evaluate the clinical outcomes and adverse events of patients. All treatments, including the selection of biological agents, dose, and duration of treatment, were determined by the treating rheumatologists. Evaluations were performed every visit after obtaining consent from each participant. In this study, we used only the biologic RA group (patients treated with biologic DMARDs or targeted synthetic DMARDs).

The present study included patients with RA who were registered in the KOBIO registry or those who had been treated with rituximab at Ajou University Hospital, but not registered with KOBIO. There was no difference of the data collection and baseline characteristics of patients between KOBIO registry and Ajou University Hospital (Supplementary Table 1). All patients fulfilled the 1987 American College of Rheumatology (ACR) or 2010 ACR/European League Against Rheumatism classification criteria for RA diagnosis (15, 16). Patients with a low-dose rituximab regimen for retreatment were excluded, considering that other regimens may result in biassed outcomes. A total of 82 patients registered in the KOBIO from its launch to 2020 (*n* = 55) and patients treated at Ajou University Hospital between 1999 and 2020 (*n* = 27) were included.

The data collection form and study protocol for current study was approved by the institutional review board of Ajou University Hospital (AJIRB-MED-MDB-21-055) or local ethics committees at each participating centre, and was conducted in compliance with the principles of the Declaration of Helsinki. All patients provided written consent to participate in the registry.

### Data Collection and Assessment of Disease Activity

Medical information was collected through data uploaded to the KOBIO web server (http://www.rheum.or.kr/kobio/). At the time of registration, individual investigators at each centre obtained information through structured interviews or using medical chart records including clinical information, laboratory tests, and radiologic imaging. Data for each patient were updated annually using a standardised case report form, and all data are transferred to the web server. Clinical information, such as age, sex, body mass index, alcohol consumption, smoking habits, extra-articular manifestations, previous or current medications, and concomitant diseases, was collected primarily from health questionnaires and interviews. Laboratory tests included rheumatoid factor (RF), anti-citrullinated protein antibody (anti-CCP Ab), erythrocyte sedimentation rate (ESR), and C-reactive protein (CRP). All radiographs were evaluated by radiologists, and bone erosion was defined as the presence of erosion of at least one proximal interphalangeal joint, metacarpophalangeal joint, wrist, and metatarsophalangeal joint on plain radiographs of the hand and foot. Disease activity was evaluated according to the number of tender and swollen joints, visual analogue scales for pain, patient's and physician's global assessment, and Disease Activity Score in 28 joints (DAS28)-ESR and DAS28-CRP.

### Rituximab Protocol

All patients received 1,000 mg of rituximab intravenously on days 1 and 15 according to the standard regimen for RA as the first cycle of treatment with rituximab (17). All patients were evaluated for disease activity and adverse events 4 months after the date of starting rituximab according to Korean National Health Insurance reimbursement criteria. And it was evaluated to be effective in 4-month evaluation, and if the disease worsened again, it could be re-administered after 6 months. Further cycles were repeated with the same regimen in patients with physician-confirmed aggravation of disease activity. Previous or current concomitant csDMARDs, corticosteroids, number of previous biologic agents, dates of onset and withdrawal, and treatment intervals were also collected. In case of treatment failure, the reasons such as lack of efficacy, adverse events, and others, were also identified. As for rituximab's efficacy, all patients are evaluated for disease activity and adverse events 4 months after the date of starting rituximab according to Korean National Health Insurance reimbursement criteria. And it is evaluated to be effective in 4-month evaluation, and if the disease worsens again, it could be re-administered after 6 months.

### Statistical Analysis

The baseline characteristics were analysed using descriptive statistics, and data were presented as the mean ± standard deviation. Categorical variables were compared using the chi-square test or Fisher's exact test, while continuous variables were compared using the independent *t*-test. Survival curves of persistence on rituximab were generated using the Kaplan-Meier method. Univariate and multivariate linear regression analyses were used to determine the variables associated with extending the time interval between retreatment courses. Binary logistic regression analysis was performed to identify the risk factors of treatment failure. The results of linear regression analyses were expressed as β coefficients, while those of logistic regression analyses were expressed as odds ratios (OR) with 95% confidence intervals (CI). All statistical analyses were conducted using SPSS version 25.0 (IBM Corporation, Armonk, NY, USA). *P*-values <0.05 were considered statistically significant.

## Results

### Clinicodemographic Patient Characteristics

The mean age at the first rituximab cycle was 55.2 ± 13.4 years, and almost all patients were female (81.7%). The clinicodemographic patient characteristics are shown in Table 1. The most common comorbidity was hypertension (31.7%). The mean disease duration was 7.9 ± 6.0 years. There were 74 (90.2%) patients who were RF positive and 55 (67.1%) patients who were anti-CCP Ab positive. The mean DAS28-ESR and DAS28-CRP were 5.87 ± 1.02 and 4.83 ± 1.12, respectively. Majority of the patients had received csDMARDs before receiving rituximab, with 56 (68.3%) patients having at least two csDMARDs. The most commonly used csDMARDs were MTX (91.5%), followed by leflunomide (32.9%) and sulfasalazine (25.6%), respectively. All patients, except 3 patients, were taking corticosteroids, and the mean corticosteroid dose was 5.61 ± 3.57 mg prednisone-equivalent. There was little change in the number of patients using csDMARDs during concomitant treatment at the time of the first rituximab cycle, but there was a difference in the number of medications. Most of the patients were taking one csDMARD, with 44 patients (53.7%) and 33 patients (40.2%) taking at least two csDMARDs. The proportion of patients taking corticosteroids was approximately the same, and the mean dose was 4.63 ± 2.85 mg prednisone-equivalent, which was lower than the dose before receiving rituximab. In total, 80 of the 82 patients had previously experienced other biologic agents and the median number of prior biologic agents was two. Among them, 55 patients (68.8%) received two or more anti-tumour necrosis factor (anti-TNF) agents.

**Table 1:** Baseline characteristics of patients with RA at time of first cycle of rituximab.

**Variable**	**RA patients (*n* = 82)**
Demographics	
Age, mean (years)	55.2 ± 13.4
Sex	
Female, N. (%)	67 (81.7)
Male, N. (%)	15 (18.3)
BMI, mean	22.9 ± 3.96
Smoking, N. (%)	16 (19.5)
Alcohol, N. (%)	7 (8.5)
Comorbidities, N. (%)	
Diabetes mellitus	7 (8.5)
Hypertension	26 (31.7)
Cardiovascular disease	1 (1.2)
Cancer	6 (7.3)
Disease status	
Disease duration (years)	7.88 ± 5.97
RF positivity, N. (%)	74 (90.2)
Anti-CCP Ab positivity, N. (%)	55 (67.1)
Tender joint count	10.3 ± 7.46
Swollen joint count	7.6 ± 5.53
ESR, mm/hr	58.9 ± 30.4
CRP, mg/dL	3.65 ± 7.76
DAS28-ESR	5.87 ± 1.02
DAS28-CRP	4.83 ± 1.12
Patient pain intensity, VAS (mm)	57.8 ± 21.0
Radiographic erosions, N. (%)	44 (53.7)
RA associated ILD, N. (%)	7 (8.5)
Medication	
Previous treatments	
Prior use of methotrexate, N. (%)	75 (91.5)
Prior use of sulfasalazine, N. (%)	21 (25.6)
Prior use of leflunomide, N. (%)	27 (32.9)
Prior use of csDMARDs, N. (%)	79 (96.3)
One csDMARD received, N. (%)	23 (28.0)
Two or more csDMARDs received, N. (%)	56 (68.3)
Corticosteroid use before rituximab treatment, N. (%)	79 (96.3)
Dosage, mean, mg/day (prednisone-equivalent)	5.61 ± 3.57
Concomitant treatments	
Methotrexate, N. (%)	66 (80.5)
Sulfasalazine, N. (%)	3 (3.7)
Leflunomide, N. (%)	14 (17.1)
Number of csDMARDs used, N. (%)	77 (93.9)
One csDMARD received, N. (%)	44 (53.7)
Two or more csDMARDs received, N. (%)	33 (40.2)
Corticosteroid use after rituximab treatment, N. (%)	76 (92.7)
Dosage, mean, mg/day (prednisone-equivalent)	4.63 ± 2.85
Prior use of biologic agents, N. (%)	80 (97.6)
Number of prior biologic agents, median (IQR)	2 (2, 3)
Prior use of ≥ 2 anti-TNF agents, N. (%)	55 (67.1)
Originator, N. (%) (vs. biosimilar)	77 (93.9)

*RA, rheumatoid arthritis; BMI, body mass index; RF, rheumatoid factor; Anti-CCP Ab, anti-citrullinated protein antibody; ESR, erythrocyte sedimentation rate; CRP, C-reactive protein; DAS, disease activity score; VAS, visual analogue scale; ILD, interstitial lung disease; csDMARDs, conventional synthetic disease modifying anti-rheumatic drugs; IQR, inter-quartile range; TNF, tumour necrosis factor*.

### Treatment Outcomes

**Figure 1:**
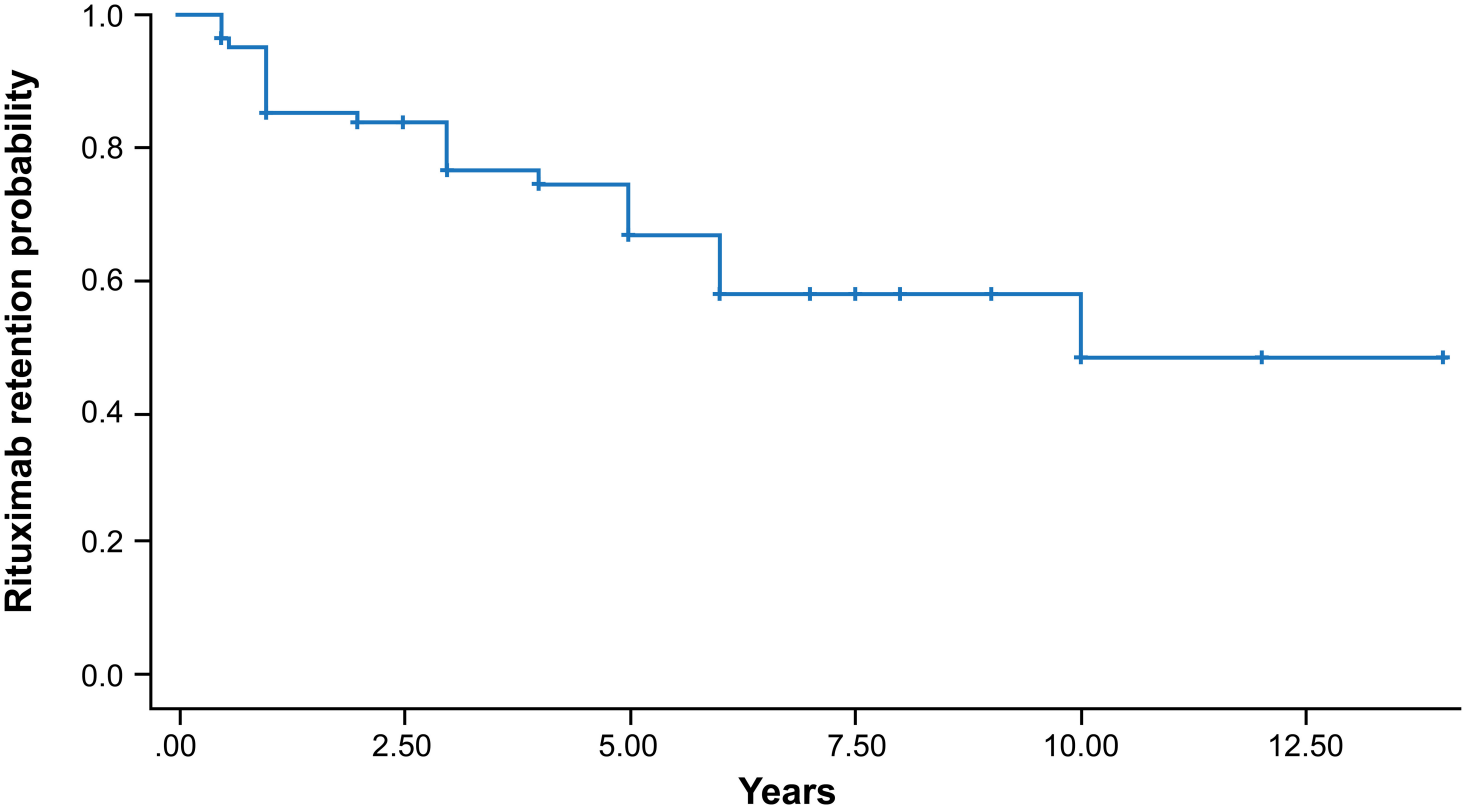
Kaplan-Meier survival curve of the persistence of rituximab in patients with RA. The cumulative persistence rates were 97.5, 89.1, 84.8, 77.8, and 72.4% every year until 5 years after the first cycle of rituximab.

The treatment outcomes after the mean follow-up of 46.1 months are shown in Table 2. Sixty-seven patients sustained treatment without failure, and 10 of them achieved biologic-free remission after the first cycle. Biologic-free remission was defined as a state in which low disease activity was maintained only with csDMARDs without the use of biologic agents after the first cycle of rituximab. In the remaining 57 patients, the retreatment schedule was adjusted according to the judgement of the physician based on disease activity. The median number of rituximab cycles was 2, and the mean time interval of the retreatment courses was 16.3 ± 8.7 months. The probability of persistence for rituximab according to Kaplan-Meier analysis is presented in Figure 1. In total, 97.5, 89.1, 84.8, 77.8, and 72.4% of the patients continued rituximab each year until 5e years after the first cycle of rituximab. Each persistence rate was calculated as the percentage of patients who were maintained in the population, excluding those with follow-up loss or a short follow-up period. Treatment failure occurred in 15 patients (18.3%). The most common reason for rituximab discontinuation was lack of efficacy [7 (46.7%) patients], followed by death after rituximab administration [5 patients (33.3%)]. The causes of death were infection and malignancy. The other reasons for discontinuation were adverse effects (13.3%) and the patient's decision (6.7%).

**Table 2:** Treatment outcome of rituximab in patients with RA.

**Variable**	**RA patients (*n* = 82)**
Total follow-up period from the first cycle, mean (months)	46.1 ± 37.5
Achieving biologic-free remission after first cycle, N. (%)	10 (12.2)
Patients still receiving rituximab at end of follow-up, N. (%)	57 (69.5)
Number of retreatment courses, mean	2.56 ± 2.31
Time interval between two courses, mean (months)	16.3 ± 8.56
Treatment persistence every year after first cycle, N. (%)	
1 (*n* = 79)	77 (97.5)
2 (*n* = 55)	49 (89.1)
3 (*n* = 46)	39 (84.8)
4 (*n* = 36)	28 (77.8)
5 (*n* = 29)	21 (72.4)
Treatment failure, N. (%)	15 (18.3)
Lack of efficacy, N. (%)	7 (46.7)
Adverse effect, N. (%)	2 (13.3)
Physician/patient decision, N. (%)	1 (6.7)
Death, N. (%)	5 (33.3)

*RA, rheumatoid arthritis*.

### Predictive Factors of Good Response to Rituximab

We analysed the clinical factors associated with achieving biologic-free remission after the first cycle of rituximab (Table 3) and found that only the previous use of two or more anti-TNF agents was significant. Disease activities, comorbidities, type or number of concomitant csDMARDs, and the corticosteroid dose did not significantly affect the achievement of biologic-free remission. Multivariable linear regression analysis of the factors that extend the time interval between the retreatment courses showed that prior (β = 5.386; 95% CI: 0.86–9.911, *p* = 0.021) or concomitant use of two or more csDMARDs (β = 4.672; 95% CI: 0.089–9.255, *p* = 0.046) and concomitant use of corticosteroids (β = 7.602; 95% CI: 0.924–14.28, *p* = 0.026) were significant factors (Table 4).

**Table 3:** Clinical characteristics of patients who achieved and did not achieved biologic-free remission after first cycle.

**Variable**	**Achieved (*n* = 10)**	**Not achieved (*n* = 72)**	***P* value**
**Demographics**			
Age, mean (years)	51.1 ± 18.0	55.73 ± 12.7	0.449
Sex, female, N. (%)	8 (80.0)	59 (81.9)	0.882
BMI, mean	23.5 ± 3.25	22.8 ± 4.06	0.648
Smoking, N. (%)	4 (40.0)	12 (16.7)	0.083
Alcohol, N. (%)	2 (20.0)	5 (6.9)	0.169
Comorbidities, N. (%)			
Diabetes mellitus	1 (10.0)	6 (8.3)	0.861
Hypertension	2 (20.0)	24 (33.3)	0.399
Cardiovascular disease	0 (0)	1 (1.4)	0.709
Cancer	2 (20.0)	4 (5.6)	0.102
**Disease status**			
Disease duration (years)	9.53 ± 5.19	7.65 ± 6.06	0.352
RF positivity, N. (%)	8 (80.0)	66 (91.7)	0.175
Anti-CCP Ab positivity, N. (%)	6 (60.0)	49 (68.1)	0.345
DAS28-ESR	5.81 ± 1.0	5.87 ± 1.03	0.858
DAS28-CRP	4.91 ± 0.81	4.82 ± 1.16	0.827
Radiographic erosions, N. (%)	5 (50.0)	39 (54.2)	0.806
RA associated ILD, N. (%)	1 (10.0)	6 (8.3)	0.861
**Medication**			
Previous treatments			
Prior use of methotrexate, N. (%)	10 (100)	65 (90.3)	0.306
Prior use of leflunomide, N. (%)	4 (40.0)	23 (31.9)	0.614
Prior use of sulfasalazine, N. (%)	5 (50.0)	16 (22.2)	0.061
Two or more csDMARDs received	8 (80.0)	48 (66.7)	0.399
CS dose before rituximab treatment, mean, mg/day (prednisone-equivalent)	4.89 ± 3.75	5.70 ± 3.75	0.525
Concomitant treatments			
Methotrexate, N. (%)	7 (70.0)	59 (81.9)	0.375
Leflunomide, N. (%)	1 (10.0)	13 (18.1)	0.528
Sulfasalazine, N. (%)	1 (10.0)	2 (2.8)	0.275
Two or more csDMARDs received	5 (50.0)	28 (38.9)	0.505
CS dose after rituximab treatment, mean, mg/day (prednisone-equivalent)	3.25 ± 2.06	4.83 ± 2.90	0.101
Number of prior biologic agents,	2 (2, 3)	2 (2, 3)	0.899
median (IQR)			
Prior use of ≥2 anti-TNF agents, N. (%)	10 (100)	45 (62.5)	0.019
Originator, N. (%) (vs. biosimilar)	1 (10.0)	4 (5.6)	0.584

*BMI, body mass index; RF, rheumatoid factor; Anti-CCP Ab, anti-citrullinated protein antibody; DAS, disease activity score; ESR, erythrocyte sedimentation rate; CRP, C-reactive protein; VAS, visual analogue scale; RA, rheumatoid arthritis; ILD, interstitial lung disease; csDMARDs, Conventional synthetic disease modifying anti-rheumatic drugs; CS, corticosteroid; IQR, inter-quartile range; TNF, tumour necrosis factor*.

**Table 4:** Factors related to extending the time intervals between rituximab treatment.

**Variable**	**β coefficient**	**95% CI**	***P* value**
Age	−0.068	−0.26–0.124	0.481
BMI	−0.274	−0.835–0.288	0.333
Smoking	−1.914	−8.495–4.668	0.563
Alcohol	−5.215	−17.598–7.167	0.402
Disease duration	0.114	−0.272–0.5	0.558
RF positivity	2.998	−6.014–12.01	0.508
Anti-CCP Ab positivity	−3.062	−12.537–6.414	0.519
DAS28-ESR	0.263	−1.859–2.386	0.804
DAS28-CRP	−0.273	−2.147–1.602	0.772
Patient pain intensity, VAS (mm)	0.058	−0.057–0.173	0.319
Radiographic erosions	4.309	−0.127–8.746	0.057
RA associated ILD	−0.2	−8.306–7.906	0.961
Prior use of methotrexate	3.669	−3.737–11.075	0.325
Prior use of leflunomide	1.257	−3.744–6.258	0.616
Prior use of sulfasalazine	−0.26	−6.07–5.55	0.929
Prior use of csDMARDs yes (vs. no)	2.057	−8.197–12.312	0.689
Prior use of csDMARDs ≥ 2	5.386	0.86–9.911	0.021
Prior use or corticosteroid	3.246	−9.185–15.678	0.603
Prior use of corticosteroid dose	−0.172	−0.753–0.409	0.555
Concomitant use of methotrexate	5.194	−0.445–10.833	0.07
Concomitant use of leflunomide	2.841	−3.716–9.398	0.389
Concomitant use of sulfasalazine	−5.377	−22.783–12.029	0.538
Concomitant use of csDMARDs yes (vs. no)	1.648	−7.318–10.614	0.714
Concomitant use of csDMARDs ≥ 2	4.672	0.089–9.255	0.046
Concomitant use of corticosteroid yes (vs. no)	7.602	0.924–14.28	0.026
Concomitant use of corticosteroid dose	−0.317	−1.133–0.499	0.44
Prior use of biologic agent yes (vs. no)	−0.744	−13.205–11.717	0.905
Prior biologic agent number	−3.514	−9.246–2.219	0.225
Prior use of ≥ 2 anti-TNF agents	−0.897	−5.696–3.901	0.709
Originator (vs. biosimilar)	−3.147	−19.588–13.294	0.704

*BMI, body mass index; RF, rheumatoid factor; Anti-CCP Ab, anti-citrullinated protein antibody; DAS, disease activity score; ESR, erythrocyte sedimentation rate; CRP, C-reactive protein; VAS, visual analogue scale; RA, rheumatoid arthritis; ILD, interstitial lung disease; csDMARDs, Conventional synthetic disease modifying anti-rheumatic drugs; TNF, tumour necrosis factor*.

### Factors Associated With Treatment Failure

The results of the logistic regression analysis for the risk factors of rituximab failure are described in Table 5. Univariate logistic regression analysis indicated that anti-CCP Ab positivity was significantly associated with treatment failure, with an OR of 0.157 (95% CI: 0.028–0.875, *p* = 0.035). This means that the probability of failing rituximab treatment is 0.15 times or 85% lower in patients with anti-CCP Ab than in patients without anti-CCP Ab. After multivariate analysis, anti-CCP Ab positivity remained as an independent factor associated with treatment failure of rituximab (OR, 0.184; 95% CI: 0.031–0.709, *p* = 0.016). Bone erosions, the presence of interstitial lung disease, concomitant medications, and the use of biosimilars did not influence treatment failure.

**Table 5:** Binary logistic regression analysis of risk factors associated with treatment failure in patients with RA after treatment with rituximab.

**Variable**	**Univariate**		**Multivariate**	
	**Odds ratio (95% CI)**	**P value**	**Odds ratio (95% CI)**	***P* value**
Age	1.021 (0.977–1.067)	0.365		
BMI	0.987 (0.854–1.140)	0.857		
Female gender	0.873 (0.213–3.578)	0.850		
Smoking	1.667 (0.453–6.138)	0.442		
Alcohol	3.937 (0.78–19.881)	0.097		
Disease duration	0.998 (0.908–1.098)	0.971		
RF positivity	1.4 (0.156–12.578)	0.764		
Anti-CCP Ab positivity	0.157 (0.028–0.875)	0.035	0.184 (0.031–0.709)	0.016
DAS28-ESR	1.042 (0.598–1.815)	0.884		
DAS28-CRP	1.241 (0.739–2.082)	0.414		
Patient pain intensity; VAS (mm)	1.01 (0.983–1.039)	0.47		
Radiographic erosions	2.833 (0.819–9.796)	0.1		
RA associated ILD	0.726 (0.081–6.523)	0.775		
Prior use of csDMARD ≥ 2	2.091 (0.536–8.163)	0.288		
Prior use of corticosteroid dose	0.996 (0.848–1.170)	0.962		
Concomitant use of csDMARD ≥ 2	1.38 (0.447–4.259)	0.576		
Concomitant use of corticosteroid dose	1.177 (0.979–1.416)	0.083		
Prior biologic agent number	1.049 (0.751–1.467)	0.778		
Prior use of ≥ 2 anti-TNF agents	0.486 (0.155–1.523)	0.216		
Originator (vs. biosimilar)	0.118 (0.117–10.852)	0.919		

*BMI, body mass index; RF, rheumatoid factor; Anti-CCP Ab, anti-citrullinated protein antibody; DAS, disease activity score; ESR, erythrocyte sedimentation rate; CRP, C-reactive protein; VAS, visual analogue scale; RA, rheumatoid arthritis; ILD, interstitial lung disease; csDMARDs, Conventional synthetic disease modifying anti-rheumatic drugs; TNF, tumour necrosis factor*.

## Discussion

On-demand rituximab administration has been reported to achieve outcomes similar to fixed-interval administration. In this study, 57 patients among the 82 patients were treated with on-demand rituximab administration without failure, and 10 of them achieved biologic-free remission after the first cycle. The mean time interval of the retreatment courses was 16.3 ± 8.7 months. Rituximab has a longer-lasting effect on the host immune system than other biologic agents approved for the treatment of RA owing to its depletion of peripheral B cells from about 90% to almost 100% (18). However, although the long-lasting effect is highly advantageous for patients who prefer a convenient lifestyle, there are concerns that early use of new biologic agents may pose additional safety risks even if the effect of rituximab is insufficient.

Given that it is difficult to cope with insufficient efficacy, it is necessary to select subjects who are predicted to be good responders to rituximab. As such, the significance of this report is emphasised as, to our best knowledge, it is the first to evaluate the treatment response to rituximab using the nationwide registry in Korea. In our study, 72.4% of patients with RA continued rituximab after 5 years, indicating higher effectiveness and tolerability than previously reported in other cohorts. The rate of persistence in most previous reports ranged from 50-60% after 4 years of rituximab (19–21). The reason for this trend is unclear because there are no differences in disease activity, seropositivity, or number of prior biologic agents between our study and other studies (19, 20).

In the present study, the time interval for patients maintaining rituximab was significantly longer than the fixed retreatment schedule of 6 months. Retreatment was performed on demand rather than on a fixed schedule, as the retreatment schedule in Korea depends highly on the Korean National Health Insurance (KNHI) reimbursement criteria. Unlike other biologic agents that are administered at regular intervals even without flare, rituximab is reimbursed only at the time of flare at least 6 months after administration of the previous course under the KNHI system. A total of 12% of patients in this study achieved biologic-free remission after 1 cycle of rituximab. Given that existing data are mainly on patients who achieved biologic-free remission following treatment with anti-TNF agents, there is limited evidence on whether biologic-free remission is sustained after the discontinuation of rituximab (22).

In a previous study, the sustained rate of biologic-free remission ranged from 14 to 60% within a short follow-up period of 2 years, and thus further studies to select the most appropriate treatment strategies are needed (23). Compared with previous studies, our study is advantageous in that it includes a fairly long follow-up period, and we found a novel result that the use of two or more anti-TNF agents prior to rituximab is the only significant factor influencing biologic-free remission. This is in contrast to previous results that a higher number of biological agents prior to rituximab experience leads to a shorter duration of the clinical response (24, 25). A study by La et al. (26) verified that prolonged exposure to anti-TNF agents could increase B-cell survival factors to induce resistance to rituximab, and this is related to the overall duration of previous anti-TNF agents, rather than the number of anti-TNF failures. In summary, instead of long-term maintenance, the anti-TNF agent should be switched in patients who do not adequately respond to the treatment.

Further, other biologic agents with a different mechanism of action, such as rituximab, should be selected if the patient does not respond to treatment with two or more anti-TNF agents. Furthermore, RF negativity, not smoking and minimal radiographic damage which have been proven to be related to biologic free remission in previous studies, have not shown significant results in this study (27). The lack of significance of baseline disease characteristics for predicting biologic-free remission after treatment implies that other factors such as genetic differences in drug metabolism may affect the response to rituximab (28, 29).

Increasing evidence shows that on-demand, rather than fixed regular retreatment, is a reasonable schedule for long-term maintenance treatment of rituximab in patients with RA (30). The time interval for the average rituximab treatment course in our patients was 16 months, which was longer than in published literature (12, 20, 31). In several real-life observational studies, the average response duration of rituximab ranged from 7.8 months to 13 months. Observational studies also demonstrated that fixed regular retreatment and on-demand retreatment with rituximab showed comparable efficacy in patients who had a good response after the first cycle of the standard regimen (30). As such, on-demand retreatment is a more favourable option with respect to safety and cost saving than fixed regular retreatment. Several studies on low-dose rituximab as retreatment to reduce side effects have proven the non-inferiority of its efficacy; however, the on-demand retreatment of low-dose rituximab has insufficient efficacy (32, 33).

Considering the current tendency to pursue on-demand retreatment globally and the convenience of extending the retreatment time intervals, it is reasonable to find ways to extend the time interval between retreatment courses rather than attempting to reduce the dose. Our study identified that prior or concomitant use of two or more csDMARDs and concomitant use of corticosteroids are associated with the extension of the time interval. Previous studies have reported that concomitant treatment with csDMARDs improves the clinical response of rituximab, and most of these studies are on rituximab in combination with a single csDMARD (34–36). To our best knowledge, we are the first to report that the concomitant use of two or more csDMARDs and/or corticosteroids with rituximab plays a critical role in maintaining clinical good responses for long periods. It is quite noteworthy in that it has an acceptable safety profile when compared to concomitant use of csDMARD monotherapy (data not shown). This finding indicates that maintaining low disease activity to prevent disease flare following rituximab treatment is important for extending the time interval between treatment courses. The persistent rates of rituximab were high in our cohort owing to the high proportion of patients taking concomitant csDMARDs or corticosteroids.

The treatment failure rate in this study was 18.3%, which is lower than that in other studies (19, 20). However, the most common reasons for treatment failure (i.e., lack of efficacy, death, and adverse events) were similar to those in previous studies (19). Binary logistic regression analysis showed that only anti-CCP Ab positivity is a significant associated factor of treatment failure. RA patients who are RF or anti-CCP Ab positive are more likely to respond better to rituximab treatment than autoantibody-negative patients. However, studies on each antibody as independent factors have reported conflicting results (24, 37–39). Some studies reported that anti-CCP Ab positivity was associated with a good response and that higher anti-CCP Ab titres predict good responses (40, 41). Meanwhile, other study reported that it is RF positivity, rather than anti-CCP Ab positivity, that is related to the good response to rituximab (25). Thus, the most reliable antibody to predict treatment response is yet to be established.

In addition, other factors, such as the B cell phenotype, have been recently reported to influence the treatment response to rituximab (42, 43). Plasmablasts, for example, was supplemented CD20 positive B cells despite being CD20 negative, becoming a potential biomarker for identifying B cell depletion after treatment with rituximab (44). However, except for anti-CCP Ab, no useful biomarkers predictive of treatment failure have been identified. Although factors other than autoantibody positivity may affect treatment failure in rituximab, rituximab should not be considered as the primary treatment option in autoantibody-negative patients.

The strength of our study is that to our best knowledge, it is the first to analyse the factors that extend the time interval during on-demand retreatment with rituximab in RA patients with a good clinical response. In addition, this is the first study to investigate the treatment outcomes of rituximab in RA patients, using data from a nationwide registry. However, our study also has some limitations. First, the observational study design can lead to an underestimation of events by relying on passive reporting such as adverse events and deaths. Further, the rate of loss to follow-up is also higher than in clinical trials. Second is the possibility of selection bias from the assignment of biological agents because the decision to use rituximab was made by the treating rheumatologist. Finally, the data from the KOBIO registry are not representative of the entire population of RA patients treated with rituximab. Given that data were mainly from outpatients, only a small portion of the patients may have been included in the registry because national guidelines require admission for the intravenous administration of biological agents. This limitation may be overcome with recruitment of additional patients from multicentre.

## Conclusions

RA patients treated with rituximab in Korea show high persistence rates. Further, the time interval between the retreatment courses was longer than in other countries. Concomitant use of two or more csDMARDs and concomitant use of corticosteroids with rituximab are significant influencing factors of extending the retreatment time interval. Importantly, an extended interval is safe and cost-efficient. These findings should be considered when selecting rituximab as a treatment for patients with RA.

## Data Availability Statement

The original contributions presented in the study are included in the article/Supplementary Material, further inquiries can be directed to the corresponding author/s.

## Ethics Statement

The studies involving human participants were reviewed and approved by the Institutional Review Board of Ajou University Hospital (AJIRB-MED-MDB-21-055). The participants provided their written informed consent to participate in this study.

## Author Contributions

J-WK, J-YJ, KS, C-HS, and H-AK contributed to the study design and data collection, analysis, and interpretation. J-WK, KS, and H-AK contributed to the data collection and/or data interpretation. All authors revised the manuscript and gave final approval for submission.

## Funding

This work was supported by a grant from the Korea Health Technology R&D Project through the Korea Health Industry Development Institute, funded by the Ministry of Health & Welfare, Republic of Korea (HI16C0992).

## Conflict of Interest

The authors declare that the research was conducted in the absence of any commercial or financial relationships that could be construed as a potential conflict of interest.

## Publisher's Note

All claims expressed in this article are solely those of the authors and do not necessarily represent those of their affiliated organizations, or those of the publisher, the editors and the reviewers. Any product that may be evaluated in this article, or claim that may be made by its manufacturer, is not guaranteed or endorsed by the publisher.

## References

[1] Aletaha D Smolen JS. Diagnosis and management of rheumatoid arthritis: a review. JAMA. (2018) 320:1360–72. 10.1001/jama.2018.13103 30285183

[2] Lin Y-J Anzaghe M Schülke S. Update on the pathomechanism, diagnosis, and treatment options for rheumatoid arthritis. Cells. (2020) 9:880. 10.3390/cells9040880 32260219 PMC7226834

[3] Tsubata T. Inhibitory B cell co-receptors and autoimmune diseases. Immunol Med. (2019) 42:108–16. 10.1080/25785826.2019.1660038 31532707

[4] Rubin SJ Bloom MS Robinson WH B. cell checkpoints in autoimmune rheumatic diseases. Nat Rev Rheumatol. (2019) 15:303–15. 10.1038/s41584-019-0211-0 30967621

[5] Tavakolpour S Alesaeidi S Darvishi M GhasemiAdl M Darabi-Monadi S Akhlaghdoust M. A comprehensive review of rituximab therapy in rheumatoid arthritis patients. Clin Rheumatol. (2019) 38:2977–94. 10.1007/s10067-019-04699-8 31367943

[6] Mok CC. Rituximab for the treatment of rheumatoid arthritis: an update. Drug Des Devel Ther. (2014) 8:87. 10.2147/DDDT.S41645 24403823 PMC3883598

[7] Cohen SB Emery P Greenwald MW Dougados M Furie RA Genovese MC. Rituximab for rheumatoid arthritis refractory to anti–tumor necrosis factor therapy: results of a multicenter, randomized, double-blind, placebo-controlled, phase III trial evaluating primary efficacy and safety at twenty-four weeks. Arthritis Rheum. (2006) 54:2793–806. 10.1002/art.22025 16947627

[8] Cohen MD Keystone E. Rituximab for rheumatoid arthritis. Rheumatol Ther. (2015) 2:99–111. 10.1007/s40744-015-0016-9 27747531 PMC4883263

[9] Trouvin AP Jacquot S Grigioni S Curis E Dedreux I Roucheux A. Usefulness of monitoring of B cell depletion in rituximab-treated rheumatoid arthritis patients in order to predict clinical relapse: a prospective observational study. Clin Exp Immun. (2015) 180:11–8. 10.1111/cei.12481 25370437 PMC4367089

[10] Mariette X Rouanet S Sibilia J Combe B Le Loët X Tebib J. Evaluation of low-dose rituximab for the retreatment of patients with active rheumatoid arthritis: a non-inferiority randomised controlled trial. Ann Rheum Dis. (2014) 73:1508–14. 10.1136/annrheumdis-2013-203480 23723317

[11] Tak P Rigby W Rubbert-Roth A Peterfy C Van Vollenhoven R Stohl W. Inhibition of joint damage and improved clinical outcomes with rituximab plus methotrexate in early active rheumatoid arthritis: the IMAGE trial. Ann Rheum Dis. (2011) 70:39–46. 10.1136/ard.2010.137703 20937671

[12] Cañamares I Merino L López J Llorente I García-Vadillo A Ramirez E. Experience with the use of rituximab for the treatment of rheumatoid arthritis in a tertiary hospital in Spain: RITAR study. J Clin Rheumatol. (2019) 25:258. 10.1097/RHU.0000000000000845 30001257 PMC6727960

[13] Buch MH Smolen JS Betteridge N Breedveld FC Burmester G Dörner T. Updated consensus statement on the use of rituximab in patients with rheumatoid arthritis. Ann Rheum Dis. (2011) 70:909–20. 10.1136/ard.2010.144998 21378402 PMC3086093

[14] KOBIO registry [Internet]. [updated 2020 (2012). Available online at: https://clinicaltrials.gov/ct2/show/NCT01965132.

[15] Arnett FC Edworthy SM Bloch DA Mcshane DJ Fries JF Cooper NS. The American Rheumatism Association 1987 revised criteria for the classification of rheumatoid arthritis. Arthritis Rheum. (1988) 31:315–24. 10.1002/art.1780310302 3358796

[16] Aletaha D Neogi T Silman AJ Funovits J Felson DT Bingham CO. Rheumatoid arthritis classification criteria: an American College of Rheumatology/European League against rheumatism collaborative initiative. Arthritis Rheum. (2010) 62:2569–81. 10.1002/art.27584 20872595

[17] Rubbert-Roth A Tak PP Zerbini C Tremblay J-L Carreño L Armstrong G. Efficacy and safety of various repeat treatment dosing regimens of rituximab in patients with active rheumatoid arthritis: results of a Phase III randomized study (MIRROR). Rheumatology. (2010) 49:1683–93. 10.1093/rheumatology/keq116 20463186 PMC2919195

[18] Hofmann K Clauder A-K Manz RA. Targeting B cells and plasma cells in autoimmune diseases. Front Immunol. (2018) 9:835. 10.3389/fimmu.2018.00835 29740441 PMC5924791

[19] Oldroyd AG Symmons DP Sergeant JC Kearsley-Fleet L Watson K Lunt M. Long-term persistence with rituximab in patients with rheumatoid arthritis. Rheumatology. (2018) 57:1089–96. 10.1093/rheumatology/key036 29566213 PMC5965076

[20] Norris-Grey C Cambridge G Moore S Reddy V Leandro M. Long-term persistence of rituximab in patients with rheumatoid arthritis: an evaluation of the UCL cohort from 1998 to 2020. Rheumatology. (2021). 93:248. 10.1093/rheumatology/keab248 33769451 PMC8824421

[21] De Keyser F Hoffman I Durez P Kaiser M-J Westhovens R Group MS. Longterm followup of rituximab therapy in patients with rheumatoid arthritis: results from the Belgian MabThera in Rheumatoid Arthritis registry. J Rheumatol. (2014) 41:1761–5. 10.3899/jrheum.131279 25128506

[22] Nagy G van Vollenhoven RF. Sustained biologic-free and drug-free remission in rheumatoid arthritis, where are we now? Arthritis Res Ther. (2015) 17:1–7. 10.1186/s13075-015-0707-1 26235544 PMC4522973

[23] Schett G Emery P Tanaka Y Burmester G Pisetsky DS Naredo E. Tapering biologic and conventional DMARD therapy in rheumatoid arthritis: current evidence and future directions. Ann Rheum Dis. (2016) 75:1428–37. 10.1136/annrheumdis-2016-209201 27261493

[24] Narvaez J Díaz-Torné C Ruiz JM Hernandez MV Torrente-Segarra V Ros S. Predictors of response to rituximab in patients with active rheumatoid arthritis and inadequate response to anti-TNF agents or traditional DMARDs. Clin Exp Rheumatol. (2011) 29:991. 22133052

[25] Quartuccio L Fabris M Salvin S Atzeni F Saracco M Benucci M. Rheumatoid factor positivity rather than anti-CCP positivity, a lower disability and a lower number of anti-TNF agents failed are associated with response to rituximab in rheumatoid arthritis. Rheumatology. (2009) 48:1557–9. 10.1093/rheumatology/kep314 19789202

[26] La D Collins C Yang H Migone T Stohl W B. lymphocyte stimulator expression in patients with rheumatoid arthritis treated with tumour necrosis factor α antagonists: differential effects between good and poor clinical responders. Ann Rheum Dis. (2008) 67:1132–8. 10.1136/ard.2007.079954 17967830

[27] van der Woude D Young A Jayakumar K Mertens BJ Toes RE van der Heijde D. Prevalence of and predictive factors for sustained disease-modifying antirheumatic drug–free remission in rheumatoid arthritis: results from two large early arthritis cohorts. Arthritis Rheum. (2009) 60:2262–71. 10.1002/art.24661 19644846

[28] Hyrich K Watson K Silman A Symmons D. Predictors of response to anti-TNF-α therapy among patients with rheumatoid arthritis: results from the British Society for Rheumatology Biologics Register. Rheumatology. (2006) 45:1558–65. 10.1093/rheumatology/kel149 16705046

[29] Stradner MH Dejaco C Brickmann K Graninger WB Brezinschek HP A. combination of cellular biomarkers predicts failure to respond to rituximab in rheumatoid arthritis: a 24-week observational study. Arthritis Res Ther. (2016) 18:1–8. 10.1186/s13075-016-1091-1 27558631 PMC4997751

[30] Teng Y Tekstra J Breedveld F Lafeber F Bijlsma J van Laar JM. Rituximab fixed retreatment versus on-demand retreatment in refractory rheumatoid arthritis: comparison of two B cell depleting treatment strategies. Ann Rheum Dis. (2009) 68:1075–7. 10.1136/ard.2008.100438 19435725

[31] Valleala H Korpela M Hienonen-Kempas T Immonen K Lähteenmäki J Uusitalo T. Long-term real-life experience with rituximab in adult Finnish patients with rheumatoid arthritis refractory or with contraindication to anti–tumor necrosis factor drugs. J Clin Rheumatol. (2015) 21:24–30. 10.1097/RHU.0000000000000166 25539430

[32] Henry J Gottenberg J-E Rouanet S Pavy S Sellam J Tubach F. Doses of rituximab for retreatment in rheumatoid arthritis: influence on maintenance and risk of serious infection. Rheumatology. (2018) 57:538–47. 10.1093/rheumatology/kex446 29267905

[33] Chandramohan P Jain A Antony G Krishnan N Shenoy P. Low dose rituximab protocol in rheumatoid arthritis—outcome and economic impact. Rheumatol Adv Pract. (2021) 21:77. 10.1093/rap/rkaa077 33605940 PMC7878847

[34] Tak PP Rigby W Rubbert-Roth A Peterfy C van Vollenhoven RF Stohl W. Sustained inhibition of progressive joint damage with rituximab plus methotrexate in early active rheumatoid arthritis: 2-year results from the randomised controlled trial IMAGE. Ann Rheum Dis. (2012) 71:351–7. 10.1136/annrheumdis-2011-200170 22012969 PMC3277723

[35] Edwards JC Szczepański L Szechiński J Filipowicz-Sosnowska A Emery P Close DR. Efficacy of B-cell–targeted therapy with rituximab in patients with rheumatoid arthritis. N Engl J Med. (2004) 350:2572–81. 10.1056/NEJMoa032534 15201414

[36] Narváez J Díaz-Torné C Ruiz JM Hernández MV Torrente-Segarra V Ros S. Comparative effectiveness of rituximab in combination with either methotrexate or leflunomide in the treatment of rheumatoid arthritis. Semin Arthritis Rheum. (2011) 41:401–5. 10.1016/j.semarthrit.2011.06.005 21862107

[37] Soliman MM Hyrich KL Lunt M Watson KD Symmons DP Ashcroft DM. Effectiveness of rituximab in patients with rheumatoid arthritis: observational study from the British Society for Rheumatology Biologics Register. J Rheumatol. (2012) 39:240–6. 10.3899/jrheum.110610 22174201

[38] Chatzidionysiou K Lie E Nasonov E Lukina G Hetland ML Tarp U. Highest clinical effectiveness of rituximab in autoantibody-positive patients with rheumatoid arthritis and in those for whom no more than one previous TNF antagonist has failed: pooled data from 10 European registries. Ann Rheum Dis. (2011) 70:1575–80. 10.1136/ard.2010.148759 21571731

[39] Khan A Scott DL Batley M. Smoking, rheumatoid factor status and responses to rituximab. Ann Rheum Dis. (2012) 71:1587–8. 10.1136/annrheumdis-2012-201758 22532635

[40] Couderc M Mathieu S Pereira B Glace B Soubrier M. Predictive factors of rituximab response in rheumatoid arthritis: results from a French university hospital. Arthritis Care Res. (2013) 65:648–52. 10.1002/acr.21865 23045227

[41] Gardette A Ottaviani S Tubach F Roy C Nicaise-Roland P Palazzo E. High anti-CCP antibody titres predict good response to rituximab in patients with active rheumatoid arthritis. Joint Bone Spine. (2014) 81:416–20. 10.1016/j.jbspin.2014.06.001 24998790

[42] Bergantini L. d'Alessandro M Cameli P Vietri L Vagaggini C Perrone A. Effects of rituximab therapy on B cell differentiation and depletion. Clin Rheumatol. (2020) 39:1415–21. 10.1007/s10067-020-04996-7 32088800

[43] Garcia-Montoya L Villota-Eraso C Yusof MYM Vital EM Emery P. Lessons for rituximab therapy in patients with rheumatoid arthritis. Lancet Rheumatol. (2020) 33:3. 10.1016/S2665-9913(20)30033-3 38273611

[44] Romão VC Vital EM Fonseca JE Buch MH. Right drug, right patient, right time: aspiration or future promise for biologics in rheumatoid arthritis? Arthritis Res Ther. (2017) 19:1–13. 10.1186/s13075-017-1445-3 29065909 PMC5655983

