# Supplementary material for: Factors Determining Retreatment Time Interval of Rituximab in Korean Patients With Rheumatoid Arthritis
Source: Front Med (Lausanne). 2021 Oct 28;8:765535. doi: 10.3389/fmed.2021.765535 (PMC8581042; doi:10.3389/fmed.2021.765535)
Supplement: Supplementary file 1 [file Table_1.docx]

| Variable | Nation registry (n = 55) | Ajou University (n = 27) | P value |
| --- | --- | --- | --- |
| Demographics |  |  |  |
| Age, mean (years) | 55.5 ± 12.5 | 54.4 ± 15.2 | 0.72 |
| Sex |  |  | 0.571 |
| Female, N. (%) | 44 (80) | 23 (85.2) |  |
| Male, N. (%) | 11 (20) | 4 (14.8) |  |
| BMI, mean | 22.7 ± 3.87 | 23.4 ± 4.18 | 0.466 |
| Smoking, N. (%) | 13 (23.6) | 3 (11.1) | 0.181 |
| Alcohol, N. (%) | 5 (9.1) | 2 (7.4) | 0.799 |
| Comorbidities, N. (%) |  |  |  |
| Diabetes mellitus | 6 (10.9) | 1 (3.7) | 0.275 |
| Hypertension | 16 (29.1) | 10 (37) | 0.47 |
| Cardiovascular disease | 1 (1.8) | 0 (0) | 0.484 |
| Cancer | 3 (5.5) | 3 (11.1) | 0.358 |
| Disease status |  |  |  |
| Disease duration (years) | 8.25 ± 6.66 | 6.93 ± 3.87 | 0.093 |
| RF positivity, N. (%) | 47 (85.5) | 27 (100) | 0.052 |
| Anti-CCP Ab positivity, N. (%) | 39 (70.9) | 16 (59.3) | 0.798 |
| Tender joint count | 9.09 ± 7.84 | 12.7 ± 6.08 | 0.041 |
| Swollen joint count | 6.96 ± 6.06 | 8.88 ± 4.04 | 0.092 |
| ESR, mm/hr | 58.4 ± 31.1 | 59.9 ± 29.8 | 0.832 |
| CRP, mg/dL | 3.71 ± 9.23 | 3.54 ± 3.43 | 0.923 |
| DAS28-ESR | 5.97 ± 1.05 | 6.07 ± 0.85 | 0.830 |
| DAS28-CRP | 4.79 ± 1.25 | 4.93 ± 0.82 | 0.596 |
| Patient pain intensity, VAS (mm) | 58.7 ± 23.6 | 55.9 ± 14.5 | 0.510 |
| Radiographic erosions, N. (%) | 31 (56.4) | 13 (48.1) | 0.231 |
| RA associated ILD, N. (%) | 4 (7.3) | 3 (11.1) | 0.561 |
| Medication |  |  |  |
| Previous treatments |  |  |  |
| Prior use of methotrexate, N. (%) | 48 (87.3) | 27 (100) | 0.054 |
| Prior use of sulfasalazine, N. (%) | 14 (25.5) | 7 (25.9) | 0.964 |
| Prior use of leflunomide, N. (%) | 15 (27.3) | 12 (44.4) | 0.122 |
| Prior use of csDMARDs, N. (%) | 52 (94.5) | 27 (100) | 0.219 |
| One csDMARD received, N. (%) | 17 (30.9) | 6 (22.2) | 0.117 |
| Two or more csDMARDs received, N. (%) | 36 (65.5) | 20 (74.1) | 0.216 |
| Corticosteroid use before rituximab treatment, N. (%) | 52 (94.5) | 27 (100) | 0.219 |
| Dosage, mean, mg/day (prednisone-equivalent) | 5.82 ± 2.49 | 5.19 ± 1.09 | 0.455 |
| Concomitant treatments |  |  |  |
| Methotrexate, N. (%) | 42 (76.7) | 24 (88.9) | 0.181 |
| Sulfasalazine, N. (%) | 2 (3.6) | 1 (3.7) | 0.988 |
| Leflunomide, N. (%) | 11 (20) | 3 (11.1) | 0.318 |
| Number of csDMARDs used, N. (%) | 51 (92.7) | 26 (96.3) | 0.528 |
| One csDMARD received, N. (%) | 30 (54.5) | 14 (51.9) | 0.886 |
| Two or more csDMARDs received, N. (%) | 22 (40) | 11 (40.7) | 0.949 |
| Corticosteroid use after rituximab treatment, N. (%) | 50 (90.9) | 26 (96.3) | 0.382 |
| Dosage, mean, mg/day (prednisone-equivalent) | 4.8 ± 2.89 | 4.12 ± 2.13 | 0.285 |
| Prior use of biologic agents, N. (%) | 53 (96.4) | 27 (100) | 0.319 |
| Number of prior biologic agents, median (IQR) | 2 (2,3) | 2 (2,3) | 0.152 |
| Prior use of ≥ 2 anti-TNF agents, N. (%) | 37 (67.3) | 18 (66.7) | 0.908 |
| Originator, N. (%) (vs. biosimilar) | 0 (0) | 5 (18.5) | <0.001 |

Supplementary table 1 Baseline characteristics of patients with RA at KOBIO registry and Ajou University hospital in the first cycle of rituximab

RA: rheumatoid arthritis, KOBIO: Korean Rheumatology Biologics registry, BMI: body mass index, RF: rheumatoid factor, Anti-CCP Ab: anti-citrullinated protein antibody, ESR: erythrocyte sedimentation rate, CRP: C-reactive protein, DAS: disease activity score, VAS: visual analogue scale, ILD: interstitial lung disease, csDMARDs: conventional synthetic disease modifying anti-rheumatic drugs, IQR: inter-quartile range, TNF: tumor necrosis factor
